# Association of Sources of Worry and Stress with Smoking in Japan: Findings from the Comprehensive Survey of Living Conditions 2010

**DOI:** 10.14789/jmj.JMJ20-OA18

**Published:** 2022-12-01

**Authors:** TOMOYO SATO, MITSUYA MAEDA, YUMI KAWATA, KOUTATSU MARUYAMA, HIROO WADA, AI IKEDA, TAKESHI TANIGAWA

**Affiliations:** 1 Department of Public Health, Juntendo University Graduate School of Medicine, Tokyo, Japan; 1 Department of Public Health, Juntendo University Graduate School of Medicine, Tokyo, Japan; 2 Laboratory of Community Health and Nutrition, Special Course of Food and Health Science, Department of Bioscience, Graduate School of Agriculture, Ehime University, Ehime, Japan; 2 Laboratory of Community Health and Nutrition, Special Course of Food and Health Science, Department of Bioscience, Graduate School of Agriculture, Ehime University, Ehime, Japan

**Keywords:** stress, worry, smoking

## Abstract

**Objectives:**

The purpose of this study is to examine which sources of worry and stress are associated with smoking behavior and if these associations are varied by gender.

**Methods:**

The anonymous data of 28,588 men and 30,813 women aged 20-79 years old in Japan were analyzed. We defined the subjects who chose “smoking everyday” as “smokers.” We also assessed 20 sources of worry and stress. Multivariable odds ratios (ORs) and 95% confidence intervals (CIs) of smoking for each source of worry and stress were calculated using logistic regression analysis.

**Results:**

We found significant association of financial stress with smoking behavior in both genders. The multivariable OR (95% CI) of smoking according to stress from financial stress was 1.22 (1.15-1.31) in men and 1.66 (1.53-1.80) in women. Moreover, the OR of smoking according to stress from human relationships and job affairs was significantly higher for women.

**Conclusion:**

We found that some sources of worry and stress were related to smoking behavior and these associations were found to be gender-specific.

## Introduction

Smoking is known as one of the coping actions for stress regardless of gender, age, and race^1)^. Previous studies have suggested that psychological distress is strongly associated with a higher prevalence of smoking behavior among adults^2-7)^ and is also associated with the risk of lifetime smoking for adolescents^8)^. Another study has suggested that reducing work stress by increasing job control, decreasing job strain, and finding a better balance between personal efforts and rewards gained from work might help smoking cessation efforts^9)^. Thus, stress management is important for smoking control.

A large Japanese cross-sectional study reported a statistically significant association between being a current smoker and severe psychological distress (SPD) for both genders; however, in terms of former smokers, the study found an association with SPD in women only^10)^. Another study using data from the Comprehensive Survey of Living Conditions (CSLC) showed that SPD was associated with people currently smoking in Japan, and the association was greater for women than for men^2)^. These suggested that there may be gender differences in the association between smoking behaviors and stress.

Women tend to have a higher risk of anxiety and depressive disorders due to the gender-based differences that may emanate from biomedical (hormonal, anatomical, and hippocampal neurogenesis), psychosocial (personality, coping, and symptom reporting), or epidemiological population-based risk factors (cultural, social, economic, and political procedures)^11-13)^. Sociocultural roles and adverse life events involving children, housing, or reproductive problems result in increased responsibilities for women, which may also cause a clear gender difference in the excess of depression onset among women^12)^.

To promote non-smoking policies, the Japanese government's Ministry of Health, Labour and Welfare (MHLW) recommended offering smoking cessation support programs through health checkups and a health guidance system, provided mainly by public health nurses. The programs would be covered by individuals' medical insurance. Although the existence of stress is interviewed, the sources of stress that relate to smoking behavior and the advice to solve these were not considered in the programs^14)^.

As the associations between smoking behaviors and stressors can differ between genders, it may be useful for effective smoking prevention programs to identify the gender-specific sources of worry and stress that could relate to smoking behaviors. However, few studies have examined the association between the sources of stress and smoking behavior using a large national dataset representative of the population in Japan. Therefore, the purpose of this study was to examine the gender- specific stress sources associated with smoking behavior among the general Japanese population.

## Methods

### Data source

We used the data collected in 2010 for the CSLC, a survey conducted by the MHLW. It is a self-administered questionnaire survey with the purposes of researching the basic subjects of living conditions such as health, medical care, welfare, pension, and income and of obtaining the basic data that are required for the planning and management of the Health, Labour and Welfare administration. This survey covers households and household members across the nation. Data on entire households (around 290,000 households) and household members (around 750,000 persons) in the 5,510 areas selected by stratified random sampling from sub-districts 1 and 8 of the 2005 Population Census's enumeration districts were used. We got official approval to use the secondary data from the Statistics and Information Department of the MHLW. We also used the data from the household and health questionnaires of the CSLC in 2010.

### Subjects

The anonymous data of 34,483 men and 36,830 women between 20-79 years of age were obtained from the MHLW. Of these, 10,742 and 1,170 people did not answer the questions regarding smoking status and experience of stress, respectively. We excluded these non-respondents from the present study. In the end, the data from 59,401 people (28,588 men and 30,813 women) aged 20-79 years old were used for this study.

### Assessment of smoking status

Regarding smoking status, participants were asked “Do you smoke?” and the options for selection were “Non-smoker,” “Smoking every day,” “Smoking occasionally,” or “Smoking before but have not smoked for more than 1 month.” We defined the subjects who chose “Smoking everyday” as “Smokers,” and the subjects who chose “Non-smokers,” “Smoking occasionally,” or “Smoking before but have not smoked for more than 1 month” as “Non-smokers.”

### Assessment of worry and stress

Similar to the previous study^15)^, the participants were asked ”Do you have worry and stress now?”, and only the persons who answered “Yes” were further asked to answer all items that matched their sources of worry and stress as follows: “Relationship with family,” “Relationship with nonfamily members,” “Related to love-making or sex,” “Marriage,” “Divorce,” “Bullying, sexual harassment,” “Lack of purpose in life,” “Lack of personal time,” “Income, finances, debts, etc.,” “Own illness or long-term care,” “Illness or long-term care of a family member,” “Pregnancy or child birth,” “Child rearing,” “Housework,” “Own studies, exams, entrance exams,” “Child's education,” “Own job,” “Job of a family member,” “House or living environment,” and “Other.”

### Other measurements

We also assessed the following measurements via the self-administered questionnaire: education level (junior high school, high school, junior college or technical upper secondary school, or university or graduate school); marital status (single, married, divorced, or widowed); and working status (Worker, Non-worker).

Regarding working status, participants were asked the following three questions: “Did you work to obtain income in May 2010?”, “Were you employed or self-employed?”, and “What was your employment status?” When the participant answered “Unemployed,” he or she was also asked whether he or she had the intention to work to obtain income. Participants who chose their working status as “Mainly doing job tasks,” “Mainly doing housework but have a job,” “Mainly doing other but have a job,” “Only attending school,” “Only doing housework,” “Other with no job.” We defined the subjects who chose “Mainly doing job tasks,” “Mainly doing housework but have a job,” “Mainly doing other but have a job” as “Worker” and those who chose “Only attending school,” “Only doing housework,” and “Other with no job” as “Non-worker.”

### Statistical analysis

Demographic characteristics according to stress status and sources of worry and stress stratified by gender are described, and chi-squared analysis was conducted. We calculated the odds ratios (ORs) and 95% confidence intervals (CIs) of smoking for each cause of worry and stress compared with those in the absence of any worry and stress using the multivariable logistic regression model. The confounding factors that are, age, education level, and marital and working statuses, included in the model as they were significantly associated with both stress and smoking status. All statistical analyses were performed using SAS ver. 9.4 software (SAS Institute Inc., Cary, USA). All probability values for statistical tests were two-sided, and values of p < 0.05 were regarded as statistically significant.

## Results

The proportion of individuals experiencing worry and stress was 48.5% (13,866/28,588) in men and 57.5% (17,719/30,813) in women, and the proportion of smokers was 36.8% (10,510/28,588) and 11.2% (3,443/30,813), respectively.

Table 1 shows the demographic characteristics according to stress status. For both genders, the persons experiencing stress were relatively younger, and had a higher proportion of being single or divorced/widowed, workers, and heavy smokers than those with no stress.

**Table 1:** Demographic characteristics according to Stress status of 59,401 people (28,588 men and 30,813 women)

	Men					Women				
	Stress		Non-Stress		p*	Stress		Non-Stress		p*
	n	(%)	n	(%)		n	(%)	n	(%)	
n	13,866	(43.9)	14,722	(52.9)		17,719	(56.1)	13,094	(47.1)	
Age(years)					<0.001					<0.001
20-29	1,886	(13.6)	2,100	(14.3)		2,369	(13.4)	1,695	(12.9)	
30-39	2,767	(20.0)	2,517	(17.1)		3,564	(20.1)	2,196	(16.8)	
40-49	2,898	(20.9)	2,360	(16.0)		3,593	(20.3)	2,005	(15.3)	
50-59	2,813	(20.3)	2,517	(17.1)		3,332	(18.8)	2,346	(17.9)	
60-69	2,185	(15.8)	3,242	(22.0)		2,900	(16.4)	2,879	(22.0)	
70-79	1,317	(9.5)	1,986	(13.5)		1,961	(11.1)	1,973	(15.1)	
Education level					<0.001					<0.001
Junior high school	1,510	(12.0)	1,852	(13.9)		1,979	(12.3)	1,739	(14.7)	
High school	5,090	(40.3)	5,723	(43.0)		6,933	(43.2)	5,364	(45.5)	
Junior college or technical upper secondary school	1,599	(12.7)	1,401	(10.5)		4,667	(29.1)	3,001	(25.4)	
University or graduate school	4,439	(35.1)	4,340	(32.6)		2,482	(15.5)	1,695	(14.4)	
Marital status					<0.001					0.07
Married	9,518	(68.6)	10,457	(71.0)		11,796	(66.6)	8,842	(67.5)	
Single	3,541	(25.5)	3,544	(24.1)		3,385	(19.1)	2,386	(18.2)	
Divorced or widowed	807	(5.8)	721	(4.9)		2,538	(14.3)	1,866	(14.3)	
Working status					<0.001					<0.001
Worker	10,944	(78.9)	11,169	(75.9)		10,252	(57.9)	7,024	(53.6)	
Non- Worker	2,922	(21.1)	3,553	(24.1)		7,467	(42.1)	6,070	(46.4)	
Smoking status					0.003					<0.001
≧21cigarettes/day	1,345	(9.7)	1,297	(8.8)		242	(1.4)	104	(0.8)	
≦20cigarettes/day	3,804	(27.4)	4,064	(27.6)		2,016	(11.4)	1,081	(8.3)	
Occasional	335	(2.4)	291	(1.98)		246	(1.4)	125	(1.0)	
Non-Smoker	8,382	(60.5)	9,070	(61.6)		15,215	(85.9)	11,784	(90.0)	

Data were shown as percent except for No. of participants. *Chi-square test.

Table 2 shows the sources of worries and stress by gender. The most prevalent causes of stress in men were “Own job (26.4%),” “Income, finances, debts, etc. (16.8%),” and “Own illness or long-term care (7.9%).” For women, the most prevalent causes were “Income, finances, debts, etc. (19.3%),” “Own job (18.2%),” and “Relationship with family (10.5%).”

**Table 2:** Sources of worries and stress by sex

	Men（28,588）		Women（30,813）		P*
	n	（%）	n	（%）	
Relationship with family	1,440	(5.0)	3,220	(10.5)	<0.001
Relationship with nonfamily members	1,927	(6.7)	3,074	(10.0)	<0.001
Related to love-making or sex	526	(1.8)	586	(1.9)	0.58
Marriage	495	(1.7)	505	(1.6)	0.38
Divorce	99	(0.4)	158	(0.5)	0.002
Bullying, sexual harassment	72	(0.3)	151	(0.5)	<0.001
Lack of purpose in life	1,512	(5.3)	1,697	(5.5)	0.24
Lack of personal time	1,272	(4.5)	1,892	(6.1)	<0.001
Income, finances, debts, etc.	4,803	(16.8)	5,958	(19.3)	<0.001
Own illness or long-term care	2,249	(7.9)	3,182	(10.3)	<0.001
Illness or long-term care of a family member	1,445	(5.1)	2,752	(8.9)	<0.001
Pregnancy or child birth	39	(0.1)	390	(1.3)	<0.001
Child rearing	222	(0.8)	1,418	(4.6)	<0.001
Housework	197	(0.7)	1,570	(5.1)	<0.001
Own studies, exams, entrance exams	374	(1.3)	466	(1.5)	0.04
Child's education	872	(3.1)	2,285	(7.4)	<0.001
Own job	7,546	(26.4)	5,598	(18.2)	<0.001
Job of a family member	593	(2.1)	1,697	(5.5)	<0.001
House or living environment	1,104	(3.9)	1,762	(5.7)	<0.001
Others	882	(3.1)	1,511	(4.9)	<0.001

*Chi-square test.

Table 3 shows the age- and multivariable-adjusted ORs and 95% CIs of smoking according to the sources of worry and stress. We observed significantly higher age-adjusted ORs of smokers for “Income, finances, debts, etc.” and “Divorce” than of non-smokers for both genders. In women, we observed significantly higher age-adjusted ORs for “Relationship with family,” “Relationship with nonfamily members,” “Related to love-making or sex,” “Bullying, sexual harassment,” “Lack of personal time,” “Child's education,” “Own job,” and “Job of a family member,” as well as significantly lower age-adjusted OR for “Child rearing.” In men, we observed significantly lower age-adjusted ORs for “Own illness or long-term care,” “Own studies, exams, entrance exams,” “Child's education,” and “Other.”

**Table 3:** Multivariable-adjusted odds ratio (OR) according to types of stress

	Men （n=28,588)						Women （n=30,813）					
	n	N of smokers (%)	Age-adjusted OR		Multivariable OR		n	N of smokers (%)	Age-adjusted OR		Multivariable OR	
Relationship with family	1,440	573 (39.8)	1.10	(0.99-1.23)	1.11	(0.99-1.24)	3,220	456 (14.2)	1.28	(1.15-1.43)	1.31	(1.17-1.46)
Relationship with nonfamily members	1,927	714 (37.1)	0.92	(0.83-1.01)	0.89	(0.81-0.98)	3,074	454 (14.8)	1.25	(1.12-1.39)	1.18	(1.06-1.32)
Related to love-making or sex	526	196 (37.26)	0.90	(0.75-1.07)	0.93	(0.78-1.12)	586	119 (20.3)	1.64	(1.33-2.02)	1.66	(1.34-2.07)
Marriage	495	196 (37.3)	0.93	(0.78-1.12)	0.96	(0.80-1.17)	505	78 (15.5)	1.15	(0.90-1.47)	1.22	(0.94-1.58)
Divorce	99	49 (49.5)	1.51	(1.01-2.25)	1.10	(0.73-1.66)	158	39 (24.7)	2.13	(1.48-3.07)	1.50	(1.02-2.20)
Bullying, sexual harassment	72	24 (33.3)	0.76	(0.46-1.24)	0.75	(0.45-1.24)	151	31 (20.5)	1.81	(1.21-2.70)	1.51	(1.00-2.28)
Lack of purpose in life	1,512	532 (35.2)	0.91	(0.81-1.01)	0.94	(0.84-1.05)	1,697	217 (12.8)	1.15	(0.99-1.34)	1.23	(1.06-1.44)
Lack of personal time	1,272	510 (40.1)	1.00	(0.89-1.12)	1.03	(0.92-1.16)	1,892	276 (14.6)	1.20	(1.05-1.37)	1.27	(1.10-1.46)
Income, finances, debts, etc.	4,803	2092 (43.6)	1.29	(1.21-1.37)	1.22	(1.15-1.31)	5,958	1104 (18.5)	1.90	(1.76-2.06)	1.66	(1.53-1.80)
Own illness or long-term care	2,249	581 (25.8)	0.67	(0.61-0.74)	0.67	(0.60-0.74)	3,182	324 (10.2)	1.11	(0.74-0.93)	1.05	(0.92-1.19)
Illness or long-term care of a family member	1,445	454 (31.4)	0.83	(0.80-1.05)	0.87	(0.77-0.98)	2,752	270 (9.8)	0.92	(0.80-1.05)	0.97	(0.85-1.11)
Pregnancy or child birth	39	10 (25.6)	0.49	(0.24-1.00)	0.55	(0.27-1.16)	390	45 (11.5)	0.75	(0.55-1.03)	0.89	(0.65-1.23)
Child rearing	222	84 (37.8)	0.83	(0.63-1.09)	0.83	(0.63-1.10)	1,418	183 (12.9)	0.84	(0.71-0.99)	0.88	(0.74-1.05)
Housework	197	62 (31.5)	0.79	(0.58-1.08)	0.77	(0.56-1.05)	1,570	199 (12.7)	1.23	(0.87-1.19)	1.08	(0.92-1.26)
Own studies, exams, entrance exams	374	80 (21.4)	0.43	(0.33-0.55)	0.59	(0.46-0.77)	466	53 (11.4)	0.80	(0.60-1.07)	0.95	(0.70-1.28)
Child's education	872	326 (37.4)	0.82	(0.71-0.95)	0.83	(0.72-0.96)	2,285	362 (15.8)	1.14	(1.01-1.29)	1.13	(0.99-1.28)
Own job	7,546	3031 (40.2)	1.00	(0.94-1.06)	1.02	(0.96-1.08)	5,598	821 (14.7)	1.17	(1.07-1.28)	1.12	(1.03-1.23)
Job of a family member	593	205 (34.6)	0.92	(0.77-1.09)	0.91	(0.76-1.08)	1,697	222 (13.1)	1.15	(1.00-1.34)	1.21	(1.04-1.40)
House or living environment	1,104	394 (35.7)	0.91	(0.80-1.03)	0.91	(0.80-1.03)	1,762	233 (13.2)	1.14	(0.98-1.31)	1.07	(0.92-1.24)
Others	882	260 (29.5)	0.72	(0.62-0.83)	0.73	(0.63-0.85)	1,511	155 (10.3)	0.89	(0.75-1.05)	0.93	(0.78-1.11)

Multivariable OR； Adjusted for Age, Education level, Marital status, and Working status.

Further adjusted for education level, marital and working statuses, some of the ORs for the sources were attenuated and did not reach statistical significance. However, the multivariable-adjusted ORs (95% CI) of smoking for “Income, finances, debts, etc.” remained statistically significant; the multivariable-adjusted ORs (95% CI) were 1.22 (1.15-1.31) in men and 1.66 (1.53-1.80) in women. In men, the multivariable-adjusted ORs for “Own illness or long-term care,” “Illness or long-term care of a family member,” “Own studies, exams, entrance exams,” “Child's education,” and “Other” also remained significant; the respective ORs (95% CI) were 0.67 (0.60-0.74), 0.87 (0.77-0.98), 0.59 (0.46-0.77), 0.83 (0.72-0.96), and 0.73 (0.63-0.85), respectively. In women, the multivariable-adjusted ORs for “Relationship with family,” “Relationship with nonfamily members,” “Related to love-making or sex,” “Divorce,” “Bullying, sexual harassment,” “Lack of purpose in life,” “Lack of personal time,” and “Own job” also remained significant; the respective ORs (95% CI) were 1.31 (1.17-1.46), 1.18 (1.06-1.32), 1.66 (1.34-2.07), 1.50 (1.02-2.20), 1.51 (1.00-2.28), 1.23 (1.06-1.44), 1.27 (1.10-1.46), and 1.12 (1.03-1.23), respectively.

## Discussion

### Summary of the results

For both genders, those who had financial stress (i.e., low family budget, debts) were associated with a higher prevalence of smoking behavior. Women with worry and stress from human relationships or their jobs were associated with a higher prevalence of smoking behavior. By contrast, men with health-related stress were associated with a lower prevalence of smoking.

### Stressor types and smoking behavior

Multifarious types of stressors are known to give rise to severe psychological distress (SPD), which may affect smoking behavior. Our study is the first to provide evidence for the association between the prevalence of smoking behavior and different sources of worry and stress, rather than just stress itself, using a large, nationally representative dataset in Japan. Our results show that associations between the sources of worry and stress with smoking behavior differed by gender, which suggests that gender-specific considerations will be needed when developing effective smoking control policies; these should be specific to each source of worry and stress.

For both genders, we found a significant association between financial problems and smoking behavior. Several studies have shown a significant association between smoking behavior and socioeconomic indicators that include an individual's economic situation, such as education, occupational status, household income, housing tenure, economic difficulties, and economic satisfaction^16-17)^. In Japan, the National Health and Nutrition Survey 2010 conducted by the MHLW revealed that the association between household income and smoking behavior was more significant in low-income families^18)^. The present study is consistent with previous studies and the government data. When we further analyzed the data by gender, we found that smoking behavior was associated with the stress related to financial problems, regardless of gender. A previous study in the US revealed that when smokers did quit, their financial stress decreased^19)^; therefore, a crucial aspect of promoting the cessation of smoking may involve the consideration of monetary expense.

Our findings also suggested that smoking behavior was associated with job stress in women. In Japan, the employment rate of women is increasing, and the proportion of women among the total working population was 43.4% in 2016^20)^. However, culturally, the burden of domestic tasks, like housework, care of the elderly, and childcare duties at home, tends to fall mainly on women. The time given for childcare and housework (in families with a child less than 6 years old) is 454 hours by women and 83 hours by men^21)^. In addition, according to the Caregiving Household Service Survey in 2000, most caregivers in the household are women (72.2% women, 19.5% men)^22)^. Married women with children often reported having poor health due to the strain of occupying multiple roles (i.e., being a wife, mother, worker, and homemaker concurrently)^23)^. Another study reported that employees who were taking care of Japanese elderly relatives were significantly associated with an increased risk of depression^24)^. The study also investigated the association between work-related stress, caregiver role, and depressive symptoms using K10, the 10- question self-rating scale developed by Kessler in the US to screen for severe psychological distress (SPD). The proportion of respondents with a high K10 score was significantly higher in women than in men. Women cope with multiple tasks, including housework, care for the elderly, and childcare duties at home, in addition to jobs outside the home; therefore, women are subjected to increased stress from various sources^24)^. Combining these results with previous studies may give some insight into the potential significance of our findings of the gender-specific sources of stress associated with smoking behavior.

Furthermore, our study showed that worry and stress emanating from interpersonal relationships were associated with a higher prevalence of smoking behavior in women. Additionally, women were more likely to suffer from multiple sources of stress in the present study. By contrast, men were not subjected to certain stressors pertaining to interpersonal relationships. As mentioned previously, the traditional social role of men in Japan is to work^20)^, and as such men often prioritize their efforts towards their jobs. On the other hand, women are traditionally expected to multitask and cope with not only their jobs but also the housework, care of the elderly, and childcare duties. Our data suggested that given these differences in societal expectations and increased caretaking demands, women were more likely to suffer stress brought on by their various interpersonal relationships.

### Strengths

In this study, we used the anonymous data derived from the CSLC, covering approximately 290 thousand households and household members (approximately 750 thousand persons) who were randomly sampled. The strength of the present study is that it is the first to use a large national dataset of 20-79 years-old adults, representative of the population in Japan, to provide results on the association of the prevalence of smoking behavior with not only the experience of stress, but also the different sources of stress. This study also provides insights into gender-specific differences in sensibility towards worry and stress.

### Limitations

Our study has several limitations. First, because the study design was cross-sectional, we could not determine any causal relationship between smoking behavior and stressors. Second, people responded to the questionnaire according to their personal view of their own levels of stress rather than an objective measure. Individuals might have had different definitions of what constituted “a lot of stress,” and their responses to a stressful event might also have varied. Third, while income level would likely be related to both smoking behavior and stressors, we could not consider income level as a variable because the data were unavailable. Fourth, we categorized “smoking occasionally” or “smoking before but have not smoked for more than 1 month” as “non-smokers” since the frequency of smoking was not asked in this survey and this might result in either an underestimate or an overestimate of the true association. Fifth, we excluded non-respondents from the present study. However, people with severe SPD or heavy smokers were considered to be more likely to decline to participate in the survey, which might have led to a selection bias. Finally, the prevalence of experiencing overall stress was higher among women than men in our present study. It could be possible that the gender difference in the prevalence of stress has influenced on our present results. However, many of previous studies have consistently revealed that women reported being more distressed than men^25)^. There is also growing evidence suggested that women and men are stressed by different types of situations^26-27)^. Nonetheless, further sensitivity study may need to be conducted by using population of no gender difference in prevalence of stress.

### Conclusion

The various sources of stress were associated with smoking behavior in a gender-specific manner. Both genders suffered from stressors related to financial issues (i.e., low family budget, debts), which correlated with smoking behaviors. Women experiencing stress associated with human relationships and work-related issues had a significantly higher OR for smoking, and men with health-related and educational stress had a significantly lower OR for smoking. To create effective tobacco control policies, it is important to be aware of the gender-specific sources of stress that may be behind an individual's smoking behavior.

## Funding

No funding was received.

## Author contributions

TS, MM, KM, AI, and TT designed and coordinated the study. TS and YK analyzed, and KM and AI provided technical assistance for the data analysis. TS interpreted the data and drafted the manuscript. All authors read and critically revised manuscript, and also approved the final manuscript.

## Conflicts of interest statement

The authors declare that they have no conflict of interest.

## References

[1] Yalcin BM, Unal M, Pirdal H, Karahan TF: Effects of an Anger Management and Stress Control Program on Smoking Cessation: A Randomized Controlled Trial. J Am Board Fam Med, 2014; 27: 645-660. 25201934 10.3122/jabfm.2014.05.140083

[2] Fujiwara M, Inagaki M, Nakaya N, et al: Smoking among adults with serious psychological distress: Analysis of anonymized data from a national cross-sectional survey in Japan. J Affect Disord, 2018; 239: 131-137. 30005326 10.1016/j.jad.2018.07.008

[3] Dube SR, Caraballo RS, Dhingra SS, et al: The relationship between smoking status and serious psychological distress: findings from the 2007 Behavioral Risk Factor Surveillance System. Int J Public Health, 2009; 54: 68-74. 19396580 10.1007/s00038-009-0009-y

[4] Forman-Hoffman VL, Hedden SL, Miller GK, Brown K, Teich J, Gfroerer J: Trends in cigarette use, by serious psychological distress status in the United States, 1998-2013. Addict Behav, 2017; 64: 223-228. 27690139 10.1016/j.addbeh.2016.09.003

[5] Hagman BT, Delnevo CD, Hrywna M, Williams JM: Tobacco use among those with serious psychological distress: Results from the National Survey of Drug Use and Health, 2002. Addict Behav, 2008; 33: 582-592. 18158218 10.1016/j.addbeh.2007.11.007 PMC2696205

[6] Hickman NJ 3rd, Delucchi KL, Prochaska JJ: Menthol use among smokers with psychological distress: findings from the 2008 and 2009 National Survey on Drug Use and Health. Tob Control, 2014; 23: 7-13. 22821797 10.1136/tobaccocontrol-2012-050479 PMC4258063

[7] Sung HY, Prochaska JJ, Ong MK, Shi Y, Max W: Cigarette smoking and serious psychological distress: A population-based study of California adults. Nicotine Tob Res, 2011; 13: 1183-1192. 21849411 10.1093/ntr/ntr148 PMC3223579

[8] Weiss JW, Palmer PH, Chou C-P, Mouttapa M, Johnson CA: Association between psychological factors and adolescent smoking in seven cities in China. Int J Behav Med, 2008; 15: 149-156. 18569133 10.1080/10705500801929825

[9] Kouvonen A, Kivimäki M, Virtanen M, Pentti J, Vahtera J: Work stress, smoking status, and smoking intensity: an observational study of 46,190 employees. J Epidemiol Community Health, 2005; 59: 63-69. 15598729 10.1136/jech.2004.019752 PMC1763376

[10] Kuriyama S, Nakaya N, Ohmori-Matsuda K, et al: Factors associated with psychological distress in a community-dwelling Japanese population: the Ohsaki Cohort 2006 Study. J Epidemiol, 2009; 19: 294-302. 19749498 10.2188/jea.JE20080076 PMC3924098

[11] Afifi M: Gender differences in mental health. Singapore Med J, 2007; 48: 385-391. 17453094

[12] Piccinelli M, Wilkinson G: Gender differences in depression. Critical review. Br J Psychiatry, 2000; 177: 486-492. 11102321 10.1192/bjp.177.6.486

[13] Marques AA, Bevilaqua MC, da Fonseca AM, Nardi AE, Thuret S, Dias GP: Gender Differences in the Neurobiology of Anxiety: Focus on Adult Hippocampal Neurogenesis. Neural Plast, 2016; 2016: 5026713. 26885403 10.1155/2016/5026713 PMC4738969

[14] Ministry of Health, Labour and Welfare: Specific Health Checkups and Specific Health Guidance, 2014. https://www.mhlw.go.jp/english/wp/wp-hw3/dl/2-007.pdf (Accessed August 18, 2020)

[15] Inaba A: Gender Differences of Stress Experience across the Lifespan: Using Japanese National Sample Data. Sociol Theory Methods, 1999; 14: 63-64.

[16] Siahpush M, Borland R: Smoking and Financial Stress. Tob Control, 2003; 12: 60-66. 12612364 10.1136/tc.12.1.60 PMC1759081

[17] Laaksonen M, Rahkonen O, Karvonen S, Lahelma E: Socioeconomic status and smoking Analysing inequalities with multiple indicators. Eur J Public Health, 2005; 15: 262-269. 15755781 10.1093/eurpub/cki115

[18] Ministry of Health, Labour and Welfare: National Health and Nutrition Survey 2010. https://www.mhlw.go.jp/stf/houdou/2r98520000020qbb.html (Accessed August 1, 2020)

[19] Siahpush M, Spittal M, Singh GK: Smoking cessation and financial stress. J Public Health, 2007; 29: 338-342. 10.1093/pubmed/fdm070 17998258

[20] Ministry of Internal Affairs and Communications. Annual Report on the Labour Force Survey 2016. https://www.stat.go.jp/english/data/roudou/report/2016/index.html (Accessed August 2, 2020)

[21] Ministry of Internal Affairs and Communications. Outline of the 2016 Survey on Time Use and Leisure Activities. https://www.stat.go.jp/english/data/shakai/2016/gaiyo.html (Accessed June 18, 2019)

[22] Ministry of Health, Labour and Welfare. Nursing care Service household survey 2000. https://www.mhlw.go.jp/toukei/list/kaigo_service.html (Accessed June 19, 2019)

[23] Arber S, Gilbert GN, Dale A: Paid employment and women's health: a benefit or a source of role strain? Sociol Heal Illn, 1985; 7: 375-400.

[24] Honda A, Date Y, Abe Y, Aoyagi K, Honda S: Work-related stress, caregiver role, and depressive symptoms among Japanese Workers. Saf Health Work, 2014; 5: 7-12. 24932413 10.1016/j.shaw.2013.11.002 PMC4048001

[25] Kornstein SG: Gender differences in depression: Implications for treatment. J Clin Psychiatry, 1997; 58: 12-18. 9427872

[26] Kessler RC, McLeod JD: Sex differences in vulnerability to undesirable life events. Am Sociol Rev, 1984; 49: 620-631. 2102495

[27] Viertiö S, Kiviruusu O, Piirtola M, et al: Factors contributing to psychological distress in the working population, with a special reference to gender difference. BMC Public Health, 2021; 21: 611. 33781240 10.1186/s12889-021-10560-y PMC8006634

